# Supplementary material for: Patterns of Intron Gain and Loss in Fungi
Source: PLoS Biol. 2004 Nov 30;2(12):e422. doi: 10.1371/journal.pbio.0020422 (PMC532390; doi:10.1371/journal.pbio.0020422)
Supplement: Table S1 — Also available at http://genes.mit.edu/NielsenEtAl/. (4.3 MB ZIP). [file pbio.0020422.st001.zip › NielsenEtAl/html/673.html]

AN5101.1.NCU07200.1.MG08041.1.FG00028.1


```
 CLUSTAL W (1.82) Multiple Sequence Alignments - Introns Inserted


Sequence 1: NCU07200.1	285 aa
Sequence 2: MG08041.1	283 aa
Sequence 3: FG00028.1	275 aa
Sequence 4: AN5101.1	311 aa
Alignment Length: 316 aa
Number Identitical Residues: 78 aa
Alignment Score (without introns) 4585


MG08041.1 	-MQINVVKTFLFALAAS-SVSALAVDT--EFRCGAPEPSEELIEASAIMAVAEAEAAANG
NCU07200.1	-MQI---KSFLLAAAAAPAALGAAVNTIKPFNCGTDAPSRQHIQMTKELAEKEAAMAAEG
FG00028.1 	-MVSKLALALFFGNALA-GLAGRALPP----RCAVADPTAEQVQHAQNLKKIESVSKVA-
AN5101.1  	MLQLRRLQDLVLMLAFL-QQTCLAVPRWGRGYCATAGPDESLKAEFRKLSALENDGIVEQ
          	 :       :.:  *        *:       *..  *  .       :   *    .  

MG08041.1 	TLAARQSALTIDTYVHVVATSTSASAGYLS0DATIQQQ~LRVMNEDYAPSGIQFVLKGTD
NCU07200.1	IMTA-QATINVNVYFHVVAASTALSDGYVT0STMINNQ~VATLNKAYAPHNIQFTLKGTD
FG00028.1 	-----AASITVDTYFHVVSSSSSK---YIS~KAKLTAQ~LKALNDAYGPNDITFKLIDTT
AN5101.1  	GSRKALEPIEIEVWFHVVSSKASG--DVVS~DGMIATQ0LSYLQDAYQNASISYRLEGVT
          	       .: ::.:.***::.::   . :: .  :  * :  ::. *   .* : * .. 

MG08041.1 	RTVNANWARDSGETAMKTALRKGTYKDLNLYF---LSSIP----------------GGIL
NCU07200.1	YTINSNWAVDGSELAMKKALRKGTYKDLNLYI---LKDL-----------------GDAL
FG00028.1 	FTTNTNWAAGNGELAMKRQLRQGDYKTLNLYFT-DVAKLGG---------------TEAL
AN5101.1  	RHINDKWARNEDELSMKDALRRGSYRTLNVYFQSDLQVLSGSESQGRLLGTSEQLSASVL
          	   * :** . .* :**  **:* *: **:*: :.:  : .:.:..   .::.. :   *

MG08041.1 	GYCYFP-ASATTST----VRLDGCTIASGTVPGGSISRFNLGKTAVHEVGHWFGLYHTFQ
NCU07200.1	GYCYFP-TSVTSKSN--DWYYDGCSILYDTLPGGSLTNYNLGHTSTHEIGHWFGLYHTFQ
FG00028.1 	GYCYFPEPNVSTGSQ--KFILDGCVIVAETVPGGTAAPYNLGGTAVHEVGHWFNLFHTFQ
AN5101.1  	GFCTLPDPSINSTSLRSSYVKDGCNVLAKTMPGGSLTHYNRGGTAIHEIGHWNGLLHTFE
          	*:* :*... .: :  :.   *** :   *:***: : :* * *: **:*** .* ***:

MG08041.1 	G---GCNGQGDLVDDTPAQASASSGCPIGRDSCPNQPGLDPIHNYMDYSDD~SCYEEFTP
NCU07200.1	G---GCSGNGDYVSDTPAQASASSGCPTGRDSCPSQPGLDPIHNYMDYSYD2TCYEEFTS
FG00028.1 	D---GCNG-GDLVADTPAQARQTSGCPARMDTCPNQAGDDPIHNYMDYSDD2VCYEEFTP
AN5101.1  	GESCSLDNEGDYIEDTPQESIPTDGCPARKDSCPGSPGVDPVHNFMDYSSD~ECYEHFTP
          	..:.. ...** : *** ::  :.***   *:**...* **:**:**** *  ***.**.

MG08041.1 	GQNARMSSMFAQFRAGK--
NCU07200.1	GQRTRMTSYWNQYRANASV
FG00028.1 	GQQTRMHSAWTAYRK----
AN5101.1  	AQVKRMRDMWFTMREGK--
          	.*  ** . :   * .
```
